# Hospital and Institutional News

**Published:** 1916-07-01

**Authors:** 


### HOSPITAL AND INSTITUTIONAL NEWS.

### CHARITY ABUSES VERSUS DEAD COMMITTEES.

ONE of the most prolific causes of public deception exploited by unscrupulous persons in the name of charity is the readiness, not to say carelessness, with which successful or otherwise prominent persons allow their names to be used as honorary committeemen or what not in connection with charitable organisations. It is understood that the Home Secretary's Committee on the control of war charities will urge in their so far unpublished report not only registration, but that the advertised committee shall be a "live" one, containing the names of those who actually conduct the work, and not mere dead-heads. Although the need for insisting on "live" committees is to be recommended in regard to war charities alone, it is to be wished that hospitals would grow ever more chary of associating "names" in purely honorary or fictitious relations with themselves. Association with hospitals should be regarded as an honour to be earned by solid usefulness and public spirit. The addition of empty names or titles can never be a source of strength, and the tradition in its favour is happily less respected every year.

### THE REGISTRATION OF WAR CHARITIES.

AT the moment of writing we must not anticipate the conclusions of the report of the Home Secretary's Committee on the control of war charities. But there is ground for believing that its broad conclusions embrace the two principles of public registration and an independent audit. Where, as in several pseudo-war charities, no balance sheet is published, or the cost of collection is out of all reasonable proportion to the amount realised for charity, experience goes to show that the "charity" is a bogus or suspect concern. It is in driving home one or other of these two test features that *Truth* has done such invaluable work for many years, and to *Truth*, in fact, for this reason must largely be given the credit for awakening that spirit of public criticism to which the appointment of the Home Secretary's Commission was due.

### OUR NATIONAL DISASTERS.

THE law of averages applies to national disasters as to everything else, and the loss of life by the sinking of great vessels, or by fire, or by individual suicide, is, in the actuarial sense, very largely calculable. It was therefore a reasonable step to approve the motion of the Public Trustee for the formation of a Council of National Disasters Relief Funds, and to accept the administration of the *Titanic*, the *Empress of Ireland*, the *Lusitania*, and any future funds which the Lord Mayor for the time being may raise. Any surplus not required for the relief of the specific objects for which any fund was raised should be available for the benefit of sufferers from subsequent disasters. The *Titanic* Fund now amounts to £331,023. In the case of the *Lusitania*, ninety-four persons are receiving allowances, but the fund stands at £12,200, or £2,000 short of the sum required to maintain them. The Prince of Wales's Fund is to be applied to for a contribution. It is a wonder that such a Council has been so long delayed.

### THE NEW VOLUME OF "BURDETT'S ANNUAL."

ONCE more "Burdett's Hospitals and Charities" is with us, though it has been delayed this year by conditions common to all printed works, conditions which have resulted in the extinction of not a few publications, and which concern production not merely on the mechanical side or in respect of cost, serious though these aspects are, but also because of the fact that, in this time of national crisis, those possessing the knowledge and skill that contribute to making a book of this calibre naturally are called upon to distribute their energy over a wider field than in the days of peace. To deal with the work in detail it may be said that the names of about 6,000 institutions of all kinds are to be found in the directory pages; and of these statistical information of an up-to-date character is tabulated, and compared in over 2,000 instances as between institutions in groups. There is to be found information in respect of the numerous hospitals in the British Dominions and Colonies and in America as well as many features, old and new, to refer to which in detail would occupy considerable space, but to select one of which attention may be called to the very useful list of military, naval, and territorial hospitals. Especial attention may profitably be given by the reader to the fifteen preliminary chapters on general charitable work, which, if taken by themselves, form an encyclopædia of information invaluable to the old hand in institutional work, and, one would imagine, indispensable to the large number of amateur recruits who, to meet the exigencies of the times, are working in the Red Cross hospitals and other fields of temporary philanthropic enterprise. "Burdett's Hospitals and Charities" is published by The Scientific Press, Limited, 28 and 29 Southampton Street, Strand, London, W.C., and the price is, as usual, 10s. 6d. net.

### BOOKS OF REFERENCE IN THE MAKING.

WHEN we receive our annual reference books—our "Whitaker," our "Burdett," or the like—we remove the paper cover, take our last year's volume from the shelf, and replace it by the new one, there to repose until the need for reference arises. This apathetic attitude is occasionally qualified by a hasty inspection of the pages, or such a reflection as, "Good gracious, how time flies!" or "The book seems larger this year"; but an honest and thorough examination is left until there is "more time." Yet the labour that one of these recognised standard books of reference represents is enormous, and the work has been directed to but one end—to provide in a handy form a large amount of information for the inquirer after knowledge, either of a general character or in some particular branch. It is only when one looks thoroughly into such a work that it is realised what expenditure of time, of human energy, and of money is entailed in its production. Take, for instance, our new "Burdett," and consider what the directory pages alone mean in the matter of preparation and checking, especially as they refer to one and the same year, and do not rely, except in indicated instances, upon stock information. The work of producing these directory pages has been done by several thousand different people, for the compilers in turn require the assistance of an executive officer of each institution to provide accurate current information. Turn next to the preliminary chapters of the Annual and look carefully at the wealth of tables of income, of expenditure, of in- and out-patient figures, of nursing cost, and of other information; each table as perfect and accurate as human patience and industry can make it, and the whole providing a series of standards whereby to judge the economy and efficiency of an institution. Examine also for a brief space the table in Chapter I., compressing into two pages the income and expenditure of 1,988 charities and producing totals which are staggering in respect of their own immensity and of all they represent in labour and philanthropy. Truly it may be said that acquaintance with our works of reference is worthy of better cultivation.

### THE SATURDAY FUND AND MILITARY SERVICE.

OWING to the working of the Conscription Act, the Hospital Saturday Fund records the loss of "thousands of its subscribers, honorary collectors, and other workers." It therefore makes a special appeal to women and to the older men to step into the places of those who have been withdrawn from civil life. Of the original seventeen members of the head-office staff the majority have been called to the Colours, and the *Journal* has published some graphic extracts from some of the letters that these men have sent home. The column in the *Journal* entitled "Thank You," contains extracts from the letters of thanks received at the head office for the surgical appliances given or partly provided by the Fund. In view of the drain on men and resources produced by the war the strength of the Fund's finances has been very gratifying.

### THE RED CROSS EMBLEM ON PRIVATE HOSPITALS.

As the result of consultation between the British Red Cross Society and the War Office, a memorandum has been issued which deals with the correct and incorrect usage of the Red Cross emblem. The fourth paragraph of this memorandum deals with the use of the Red Cross flag by auxiliary Red Cross hospitals, such as private houses which have been handed over to the military authorities for the reception of sick and wounded soldiers. Such hospitals must have written authority from the War Office, and they must fly the Union Jack alongside the Red Cross flag. It is not perfectly clear whether this means that the two flags must be flown at the same level on separate staffs, or whether they may be flown one above the other as in field hospitals. Inasmuch as the War Office offers to give an opinion as to the legitimacy of exhibiting the Red Cross flag in any given case, doubtless this point would be cleared up also for the benefit of those in doubt. The wording of the circular makes it plain that many private hospitals at present flying the Red Cross will no longer be permitted to do so, and that many which have the right to fly it will henceforth have to fly the Jack as well. The rest of the instructions issued refer to the use of the Red Cross emblem on motor-cars, uniform stores, and advertisements.

### WORKSHOP COLLECTIONS AFTER THE WAR.

WE have drawn attention in the following paragraph to the continued expansion which is attending the Saturday Fund movement in Birmingham, and the additional £2,000 which is being given from this source to the hospitals this year. Satisfaction with this result must not blind our eyes to the fact that it is due to the army of munition workers which is now concentrated in and about the city. The question then arises, Will the increased grant be maintained, or will the income again drop when the munition industry becomes nothing but a memory? Reduced grants are never popular, and it has been suggested that the individual weekly contribution be increased from one penny to three-halfpence, a move which the firm of Piggott and Co. has already initiated, and that the women's contributions should be the same as the men's. As the accommodation for women convalescents has latterly been increased, it is hoped that this change, too, may be effected. Birmingham is not the only city faced with these prospects, and its example will be of interest to all the hospitals which largely rely upon workshop collections for annual maintenance.

### THE WAR AND THE SATURDAY FUNDS.

THE executive committee of the Birmingham Hospital Saturday Fund has decided, with the delegates' permission, to raise the customary contribution to the hospitals from £10,000 to £12,000 this year. This recognition of their special difficulties is all the more welcome and generous since the Saturday Fund's activities have been considerably disorganised owing to the taking over by the military of the Droitwich institutions, to which the rheumatic patients benefited by the fund were formerly sent. After discarding as unsatisfactory a system of boarding out in cottages, another establishment at Droitwich was acquired and used for men; while another for women had to be erected close by. Even so, further provision for the growing number of convalescent patients is demanded, and consequently the demands on the Saturday Fund have grown very heavy. Nevertheless, it is hoped to beat even last year's record of £23,112.

### PHYSICAL CLINIC FOR DISABLED SOLDIERS.

A NEW clinic for wounded and disabled soldiers is to be opened for patients on July 3, with accommodation for sixty cases per day from 2 to 7 P.M. The clinic is housed at 126 Great Portland Street, and has been open for inspection by the medical profession on June 26 to 28, and by nurses on June 29. The equipment includes douches in various forms, and electrical appliances, as well as hot-air and other radiation baths. There are also installed improved types of the so-called whirl-pool bath, known in France as *eau courante*: these consist essentially of water, or air and water, in rapid motion and at high temperatures, and they prepare a limb for massage, for manipulation, and for movement. Another feature of the clinic is the mechanical apparatus, which has been obtained from Paris, and is especially designed for the treatment of military cases. Each patient will be submitted to expert examination by the honorary medical staff, and the intention is expressed of carrying on the work in consultation with the surgeons who have had primary charge of the patients. The treatment will be entirely free of charge, and will in the first instance be given to wounded and disabled officers. Probably something similar will be done for the rank and file if this new clinic turns out a success.

### AN EXTRAORDINARY ANNOUNCEMENT.

The editor of the *New Witness*, who is, if somewhat mercurial and eccentric, at least an able and honest journalist, has thought fit to announce in an editorial article the opening of an "Osteopathic War Clinic" in London for the free treatment of sick and wounded soldiers. The article reads more like an advertisement concocted by the American "osteopathist" himself than the considered opinion of an editor with any respect for himself or his readers. The claims set forth for the "treatment" are couched in the most studiously vague yet high-flown language; but perhaps the future articles, in which a "more complete description" is to be given, will be somewhat more explicit as to this mysterious treatment. We are not told whether the Yankee "osteopathist" is qualified as a medical practitioner in the United States; presumably he is not, as he is not given any medical prænomen. Since the treatment of sick and wounded soldiers is the affair of the Army Medical Department, under whose supervision all supplementary medical aid is administered, it is somewhat difficult to see whence the Osteopathic War Clinic proposes to draw its cases, unless it takes steps to secure official recognition. We commend to the *New Witness* the organisation of the new clinic at 126 Great Portland Street as an example of how to set about this kind of work; and to the public at large the "Osteopathic Clinic" as an equally striking example of how not to do it.

### CONCERTS AT THE FRONT.

Nearly a year ago an account was published in The Hospital of the work of the Concert Parties which Miss Lena Ashwell sends to France, under the auspices of the Y.M.C.A., to amuse our sick and wounded soldiers; and her cause was warmly commended to the benevolence of the British public. At that time the work done by the Concert Parties was much less extensive than it is now, and the financial needs of Miss Ashwell's organisation were correspondingly modest. Having endorsed her appeals then—amongst the first to recognise the value of her work—we do not hesitate to do so again, and to ask once more for even more financial support than she has had in the past. It is unnecessary to repeat the substance of the article which has been referred to, the more so in that Miss Ashwell's latest pamphlet reprints the pith of the matter as it was then expressed in our columns. Probably the terms of the Royal Warrant establishing the Royal Red Cross decoration preclude the bestowal of that distinction upon an Englishwoman who is not actually in the service of the Crown; but if not, we know of none with a better claim than Miss Ashwell to that recognition of loving labour on behalf of the sick and wounded in our wars.

### HOSPITAL LIFE IN LOCAL NEWSPAPERS.

Hospital news is steadily making its way into all classes of newspapers, and perhaps provincial institutions are providing more matters of interest to local publications than ever before. For instance, there has existed in the Midlands an old-established monthly journal, known as *Edgbastonia*, which circulates, we understand, in the West-end suburb of Birmingham. Always concerned with the public matters of the district, its May number is mainly devoted to local hospital affairs. It begins with an article on Miss E. M. Musson, matron of the General Hospital, Birmingham, who is now acting as principal matron of the 1st Southern General Hospital, an institution, by the way, with five branches. This is followed by an account of the way in which the amusements for the wounded are organised, and then follows an instructive article on the Birmingham and Midland Eye Hospital, the secretary of which is Mr. J. W. Pearce. It describes the growth of the hospital, and the first introduction of the ophthalmoscope in 1865. A heating installation on modern principles is badly required, and in view of the fact that the hospital devotes 40 of its 108 beds to wounded soldiers, the presentation to the institution of the £2,000 required would be a fit thankoffering which the residents of Edgbaston might very patriotically supply.

### DONCASTER'S NEW INFIRMARY SCHEME.

Owing to three fortunate bequests which amount to £21,500 the scheme for a new infirmary for Doncaster may probably be realised earlier than was thought possible. The late Mr. John Chester Coulman, of Thorne Levels, has left £6,000 to the infirmary, which was more than the estate was expected to be worth. But even more important is the hall and grounds bequeathed by the late Miss Beckett Denison for a new infirmary, which gifts are valued at £15,000, and, in addition, another £500 is adjudicated to the infirmary from the late Mrs. Geldart's provisions for charity. With such important aid the scheme for a new infirmary should be brought much nearer realisation, provided that labour and other questions can be arranged satisfactorily.

### AN OBJECTIONABLE PROCEEDING AT WESTCLIFF.

The reports in the papers of the plan whereby a hospital at Westcliff lately sent out wounded soldiers with collecting boxes to raise money on its behalf have naturally aroused indignation. Scandals of this kind arise from time to time from the fact that the need for raising money, and the belief that the more sentimental the appeal the better, leads unbalanced persons to lose all sense of decency. One would hardly have thought it necessary to point out that "begging is not the right sort of job for a man who has been injured in the service of his country," but it seems that those in charge at Overcliff Hospital badly needed the reminder of the *Daily News* on this head. The real wonder is that any wounded man was found to consent to the proposal, were it not that soldiers grow so much accustomed to doing what they are told that they do whatever it happens to be without thinking. By this time, however, we hope that an objectionable proceeding will have been completely squashed, and that those responsible will not venture any repetition.

### THE MILK SUPPLY AT HASLAR.

From the somewhat vague reply made by the Duke of Devonshire in the House of Lords to Lord Northbrook's question regarding the milk supply at Haslar Hospital, we hope that the patients will now receive a fit and proper milk. Under a recent contract the supply was found deficient in milk fat, but the contractors have been given one more chance, with a warning that the next offence will lead to the removal of their name from the Admiralty list. Perhaps the most unfortunate feature of the affair, however, was the unseemly quarrel between the hospital authorities and the County Council in regard to the inspection of the milk. The Admiralty officials apparently refused to allow the Council to take samples except at the hospital's gates—a plan so undesirable in itself, and so suggestive of officialdom and red tape, that it only deserves ridicule. The hospital seems to have had its lesson, however, and we trust that milk inspection, as well as milk supply, will now be put on a proper public basis.

### SOME LARGE CONTINGENT AND OTHER BEQUESTS.

A series of very large actual and contingent bequests to charities and institutions is contained in the will of Mr. Charles Cox, who died at the age of eighty on April 29. The will is proved at £117,611; out of this sum there are to be paid legacies to various institutions, chiefly ecclesiastical, amounting to £20,000. Then a trust is created of £45,000 for Mr. Cox's son and the latter's wife and children: in the event of failure of issue £37,500 of this trust is devised to the French Protestant Hospital, the Poplar Hospital, and St. Mark's Hospital for Fistula, which thus stand to receive £12,500 each if Mr. Cox, junior, leaves no children. Whether these contingent bequests are or are not likely to become actual is not stated. Certain other legacies are then bequeathed, and the residue of the estate, which will amount to a very substantial sum, is divided between the City of London Hospital for Diseases of the Chest, the Queen's Hospital for Children, the Prince of Wales's Hospital, Tottenham, and the Royal London Ophthalmic Hospital. It will be seen that all these institutions are situated in the East End, within reach of Hackney, where the testator had formerly resided. The East-End hospitals are sometimes said not to do so well in legacies as those further West; this will does something to redress the balance.

### MINERS' PHTHISIS IN SOUTH AFRICA.

According to our contemporary the *South African Mining Journal*, the Report of the Select Committee on Miners' Phthisis shows evidence in its drafting of a genuine desire to recommend alterations in the existing law so as "to bring about a diminution in the intensity and prevalence of the disease." It is stated that the mines are "well but not fully" equipped with water and the necessary appliances for reducing the amount of dust, and as means of ensuring that all may be done that can be in this respect further and more frequent inspections are recommended. Systematic dust sampling is to be made compulsory, and it is also suggested that the single-shift system should be adopted in all mines except in certain exempted cases. Recent medical research would seem to indicate that the problem of the control of phthisis is not so formidable as has been anticipated, and that its total eradication from the mines, therefore, may not be the utter impossibility it was thought to be some few years ago. "There is no reason to doubt," says the Report, "that one may look to a continued steady decrease" in the prevalence of the disease which has been the bane of miners in past years.

### THIS WEEK'S DRUG MARKET.

A quiet tone prevails in the drug market, and were it not for the continued steady buying on the part of the British and Allied Governments business would be very dull indeed. Of course, the high prices tend to restrict the demand, and although in a number of cases the downward tendency is still noticeable, prices must remain enormously above the normal so long as the war lasts. Bromides are again distinctly lower, but even this further reduction has not stimulated the demand, buyers apparently holding the belief that there is still room for another drop in value. Salicylates also continue to have a downward tendency in value, but here again prices are still extremely high, being fully sixteen times the pre-war figure. Sulphonal is rather lower, but amidopyrin and barbitone have an upward tendency. Bismuth preparations are obtainable from holders of stocks at something below the prices the latter were able to obtain until recently, but such holders can still obtain premiums on makers' prices. In citric and tartaric acids there is no substantial change. Cod-liver oil is cheaper, but the value is still far too high to tempt purchasers. Menthol has a lower price tendency, and the same may be said of ipecacuanha.

